# Identification of a new binding protein in the insect-pest midgut Heliothis virescens that interacts with Cry1A toxins

**DOI:** 10.1186/1753-6561-8-S4-P94

**Published:** 2014-10-01

**Authors:** Patrícia Pelegrini, Diogo Martins-de-Sa, Jefferson Jesus, Wagner Lucena, Maria Fatima Grossi-de-Sa

**Affiliations:** 1 Plant-Pest Interaction Laboratory, Embrapa - Genetic Resources and Biotechnology; 2 Universidade de Brasília, Brasília, Brazil; 3 Universidade Católica de Brasília, Brasília, Brazil; 4 Embrapa - Cotton, Campina Grande, Brazil

## 

*Bacillus thuringiensis *crystal protein family (Cry) consists of a diverse group of proteins with activity against insects of different orders, such as the Lepidoptera members. Their primary action is to lyse midgut epithelial cells by inserting into the target membrane and forming pores. Among this group, members of Cry1A family are used worldwide for insect control, and their mode of action has been characterized in some detail, although it is not completely known. Cry1A-binding proteins detected on ligand blots of insect brush border membrane vesicles (BBMV) have been identified as members of the aminopeptidase N and cadherin families, although the relative role of the two putative receptor molecules in insects has yet to be conclusively determined. Moreover, it seems that there are other proteins in the midgut cell surface of insect-pests that can interact with Cry1Ac, leading to cell death. Therefore, in this report, we identified the gamma region of a G-protein from *Heliothis virescens *(HvgGP) as a potential receptor for Cry1Ac. Hence, using *in silico *analyses, we determined the structure of HvgGP and its interaction region with Cry1Ac. The binding sites was confirmed through Phage Display assays, using both Cry1Ac and HvgGP as templates. Fluorescence analyses indicated that HvgGP interacts with Cry1Ac in a specific region. Although the mode of action through membrane pore formation was already confirmed by several *in vivo *and *in vitro *assays, the mechanism through inhibition/activation of signalling pathways by the interaction with G protein complexes is still not clear. Considering the importance of G proteins on the activation of several signaling pathways and the role of Cry toxins in the agribussiness, we also propose a new mechanism of action for Cry1Ac, using HvgGP as the binding protein.
